# A narrative review of nursing in Saudi Arabia: prospects for improving social determinants of health for the female workforce

**DOI:** 10.3389/fpubh.2025.1569440

**Published:** 2025-08-15

**Authors:** Julie Davies, Emily Yarrow

**Affiliations:** ^1^Brunel Business School, Brunel University of London, Uxbridge, United Kingdom; ^2^Business School, Faculty of Humanities and Social Sciences, Newcastle University, Newcastle upon Tyne, United Kingdom

**Keywords:** gender equality, gender equity, healthcare, nursing, palliative care, Saudi Arabia

## Abstract

**Introduction:**

Nurses represent the backbone of global healthcare systems where women typically deliver care and men lead. The Kingdom of Saudi Arabia (KSA) highly relies on foreign female nurses despite Saudisation policies designed to localize its workforce. Yet, in the context of an ambitious 2016–30 national health transformation program to modernize public health in a high-income and rapidly developing country, intersectional inequalities persist for migrant and local nurses in KSA. There are challenges in the specialisms of palliative and end-of-life care where there is a weak public and professional understanding of caring for people with life-limiting diseases and a lack of public discourse about death and dying. In this context, we ask which social determinants of health (SDOH) might be improved for female nurses in KSA to support SDOH improvements in the general population.

**Methods:**

Based on a narrative literature review of 36 studies using Cochrane, EBSCO, PubMed, and Web of Science databases, we highlight the social determinants of health and wellbeing for both migrant nurses in PC and general population health.

**Findings:**

Four key themes emerged from the literature review related to equity, ability, compassionate support, and meaning in the workplace. We illustrate that key SDOH for female nurses in KSA include inadequate housing, labor market fragmentation; gender, pay, religious, language and racial discrimination against nurses from low and middle-income countries with a general lack of understanding about PC. In turn, we explore these factors within four main themes: equity; ability; compassionate support; and meaningful work. Workforce shortages can result in working shifts and overtime with a lack of flexible working, which in turn cause stress, burnout, precarity, low job satisfaction and high turnover. We identify gaps between national policy ambitions for modernization and everyday practices caring for the Saudi nursing workforce.

**Discussion:**

Our review is novel in exploring the social determinants of health in the healthcare workforce linked to social determinants such as housing, income, job insecurity and working life conditions, living conditions, social inclusion and norms and socioeconomic position. It contributes to our understanding of how patriarchal cultures shape institutional and subjective forms of inequality in stark contrast to the national rhetoric of female empowerment. We propose three policy recommendations to improve PC nurses’ SDOH and, in turn SDOH for the general population: (i) gender and race pay equity, (ii) human resource for workforce health and education policies using telehealth, and (iii) professional and public communication campaigns to increase respect for nurses and understanding the benefits of PC nursing as a career and as a service.

## Introduction

This literature review of 36 studies aims to understand challenges related to the SDOH for female nurses in PC in KSA. There is high reliance on overseas nurses and insufficient supply with high levels of turnover. In the context of national transformation, growing demand for PC nursing expertise and new forms of delivery outside acute hospitals amongst an ageing population, this article contributes to the literature on healthcare workforce disparities by arguing how improving nurses’ SDOH can improve overall population health.

PC is developing as a medical speciality in KSA to improve the quality of life for terminally ill patients and those with life-limiting diseases and their families through diagnosis, pain relief and support for psychological, social, and spiritual distress (1). AlMobarak (1) estimated that in 2022, 80,000–101,000 people could have benefited from PC services. KSA needs high-quality and equitable PCs services.

Importantly, non-Muslim PC nurses struggle to understand cultural and religious awareness and the family’s role in KSA (2). One study reported on Muslim patients in KSA who refused pain management for religious reasons and the importance of respecting these wishes while making efforts to comply with advance directives to relieve suffering (3) As the KSA population ages, there will be increased demands for PC nursing services in a country where there is a strong reliance on foreign nurses. Therefore, it is vital that gaps in understanding and inequalities in working conditions are examined to realize national healthcare reforms. A unique aspect of this study is the link between PC and workforce inequalities in the context of changes with increases in delivering telehealth PC and community services. Many terminally ill patients prefer to die at home (4) or in a hospice and not in hospital. Effective integrative PC is essential in healthcare systems to benefit patients with life-limiting illnesses.

During the second phase of KSA Vision 2030 and Health Sector Transformation Program (5), we seek to conceptualize gender disparities for foreign and local female Saudi nurses. Nurses form the backbone of health systems (6). It is important to understand policy implementation gaps and lived experiences. Often overlooked are the links between social determinants (7) that affect workforce wellbeing and the general population’s health, as the focus is usually on the latter. The social determinants relate to aspects such as access to affordable and decent quality health services, education, housing, basic amenities, income, social inclusion, job security and decent working life conditions.

We call for a better understanding of how interventions might be designed to address sociocultural factors and leadership development needs amongst migrant female and local Saudi nurses to improve their motivations and retention with implications for reducing gender disparities. We contribute important insights into policy implementation in developing countries’ national reforms and intersectional inequalities (8) with wider public health ramifications.

Additionally, we consider one illustrative PC case at King Faisal Specialist Hospital & Research Centre, Riyadh (KFSH&RC), which established the first PC center in the Middle East in 1992 (9). The challenge is that resource allocation is insufficient and patchy; there are few specialists in this field, and patients can have trouble accessing pain medications (9). Additionally, there are digital and geographical divides for remote regions in KSA regarding patient care and healthcare workforce deployment. Insufficient PC services and a lack of realization that PC requires specialist units and professional staffing mean that inequalities are exacerbated in the last phase of life. This is important because healthcare costs in the last year of life are typically the highest in a patient’s life (10). Working in PC causes burnout in healthcare workers (11). Different stress levels in various healthcare specialties must also be considered when considering nursing gender disparities in KSA.

SDOH, i.e., non-medical factors such as living and working conditions, socio-economic policies and systems, and social norms, can adversely affect health equity in the workplace and society more generally. KSA is the largest country geographically in the Middle East, a high-income developing nation with an ambitious Vision 2030 Health Sector Transformation Program (HSTP) to enhance innovation, financial sustainability, disease prevention, and access to high-quality healthcare. Although KSA reforms have improved women’s rights since 2019, socially constructed differences linked to gender, social status, and religion create simultaneous intersectional inequalities which can adversely affect workplace wellbeing (12) social determinants of public health (7) in KSA. The ban on women driving in the KSA ended on 24 June 2018. However, there is gender sensitivity in terms of who can access healthcare (13), significant health workforce challenges (14) and issues of equity, efficacy and productivity (15).

To make sense of gender disparities between national policy ambitions and lived realities in the context of wider societal inequities impacting SDOH, this article will explore the following two research questions:

1. What challenges influence female PC nurses’ SDOH in the context of KSA health sector transformation?
2. How might these challenges be addressed to support SDOH in the general KSA population?

This article first contextualizes the challenges for nurses in KSA. Second, the materials and methods section describes the systematic review and discusses its limitations and strengths. Four themes identified in the literature review related to equity, ability, compassionate support, and meaning in the workplace are presented. These are then discussed with key recommendations and a conclusion.

## The Saudi Arabian context

The Saudi Nurses Association was established in 2018 to improve nursing service efficiency and develop healthy working environments and core competencies. PC was first introduced in KSA in 1992, and since 2001, the Saudi Commission for Health Specialties (SCFHS) has developed specialized educational programs in PC (9). The prevalence of cancer is increasing in the Kingdom (16) and driving the need for specialist PC nurses. It is predicted that the prevalence of cancer will increase five to 10-fold by 2030 (9). Critically, the World Health Organization estimates there will be a shortage of 10 million health workers by 2030 globally (17). In 2023, more than 235,000 nursing staff were registered with the Saudi Commission for Health Specialties. Studies indicate that expatriate nurses comprise a significant majority of the KSA nursing workforce, as high as 70% in 2018 (18), mainly from India, the Philippines and Malaysia.

The population of KSA in March 2025 was 34.3 million (19). In 2024, Saudis constituted 55.6% of the total population. Non-Saudis represented 89.9% of the working-age population (15–64 years), compared with 62.7% for Saudis. Almobarak (1) estimated that in 2022, around 101,623 people required palliative care, which included an annual need for non-cancer palliative care for 20,600 patients, and a monthly estimate for palliative cancer care for 924 patients. Yet there are only seven training centers in KSA from which around 20 doctors graduate annually. In 2024, KSA had over 68 specialist PC physicians, four palliative care services, eight community palliative home care services, and 22 PC units, outpatient services, and consultation services (9) and it is notable that there are currently only a few dedicated hospice-type facilities in KSA. There are no data on the number of nurses in KSA who provide PC (20).

Challenges persist in KSA related to allocating PC healthcare workforce resources and the availability of pain relief, especially opioids, in different geographical regions (9) to improve the quality of life for patients with terminal/life-limiting diseases and their families. PC is uniquely difficult due to socio-cultural, religious, and ethical tensions, and it critically remains a context where state policies may not fully support PC (21). A survey of 710 nurses (22) exploring knowledge and attitudes towards palliative and end-of-life care found 13.4% of nurse frequently encountered cultural challenges and 21.8% experienced such challenges occasionally.

Key challenges focused on treatment preferences, disclosure of diagnoses, and, most notably, family decision-making dynamics. Unsurprisingly, the literature suggests a correlation with burnout and potential for burnout amongst palliative health care workers in the Kingdom (23). There are clear positive correlations between nurses’ quality of work life, loyalty and job performance (24). Importantly, PC in KSA must be developed in line with standards established in other parts of the world, such as those published by the European Association for PC (25) and with a nuanced understanding of Muslim patients’ needs.

There have been notable developments in the KSA nursing profession and workforce (24) driven by population growth and rapid economic growth. In the context of Vision 2030, ongoing challenges surrounding nursing shortages and expatriate labor continue to undermine national ambitions, the patient experience and expatriate nurse wellbeing (26), and social determinants of health more broadly. Yet, the impact of patriarchal culture [where male-dominated gender norms persist (27) on society remains high (28)], where women’s resistance and conformity are “shaped by the ethnopolitical, religiopolitical and sociocultural webs of meaning” p. 873 (29). Alreshidi et al. (30) highlight the need to enhance professional growth and development, leadership styles, management, wage and benefits, workloads, interpersonal relationships, housing facilities/services, and hospital facilities to improve foreign nurses’ intent to stay and reduce turnover intentions.

While there are provisions in the Saudi national budget to empower women “for a vibrant society, a thriving economy and an ambitious nation” (31), Saudi women’s socio-economic vulnerability persists (32). Although Saudi women comprise 49.99% of the total number of higher education students (33), the Saudi female labor force participation rate was 34.5% in 2023 (34). KSA was ranked 126 out of 146 countries in the WEF *Global Gender Gap Report 2024* (35), Change 60% to Sixty percent women reported they had a bank account in Saudi Arabia (36). In terms of SDOH, unsurprisingly, non-communicable diseases are higher among Saudi women with low incomes and low education (37). Financial and family challenges, mental health issues and aging are common social problems in Saudi primary health care (38). SDOH challenges in the general Saudi population include inadequate healthcare at home services (15). There is particularly a shortage of nurses in rural areas, where the quality of healthcare delivery is uneven (39).

Leadership disparities in healthcare are not only a function of policy and cultural norms but are also deeply tied to workforce development challenges. In KSA and across the Middle East, female nurses constitute the majority of frontline healthcare providers, yet they remain under-represented in decision-making roles (40). Studies have shown that transformational leadership approaches can significantly improve job satisfaction, retention, and, ultimately, patient outcomes (24, 41). However, existing competency frameworks lack structured pathways for female nurses to transition into leadership roles (42).

A lack of mentorship programs further reinforces gender disparities in leadership. Limited access to professional training and rigid workplace structures that do not accommodate work-life balance needs disproportionately affect women in the healthcare workforce(12, 43). Furthermore, policies around nationalization (Saudisation) of the workforce often overlook gendered career progression pathways, placing expatriate female nurses at an additional disadvantage (44).

In the context of Saudi Vision 2030, efforts to nationalize the healthcare workforce provide a critical opportunity to integrate gender-inclusive leadership policies that actively promote female leadership in healthcare organizations (40). Addressing these disparities requires targeted leadership development programs and policy interventions that enhance mentorship, professional training, and access to executive roles for women in healthcare (6). By embedding leadership competencies into professional development frameworks, healthcare systems can ensure that gender equity extends beyond representation to meaningful participation in strategic decision-making. Empowering women in the health sector in KSA is critical to supporting the Kingdom’s societal and economic ambitions (45).

## Materials and methods

To investigate the research questions, literature search terms included literature search terms included (“palliative care,”) (“nursing challenges”) (“foreign nurses,”) (“end of life care,”) (“Saudi Arabia,”) (“healthcare,”) (“women,”) (and “gender differences”). No regional comparisons were made because KSA represents traditional values and beliefs with the Kaaba and mosque in Mecca representing the center of the Islamic world. KSA occupies a special place and set of values in the Arab world with a different socio-cultural context for expatriates from other Gulf Cooperation Council countries such as the United Arab Emirates or Bahrain. Only academic journal articles were reviewed, including literature reviews. Doctoral theses and grey literature were excluded.

A PRISMA (Preferred Reporting Items for Systematic Reviews and Meta-Analyses) review (46, 47) was carried out of peer-reviewed academic literature from 2010 to January 2025 to ensure rigor and relevance to evaluate literature related to the research questions about SDOH challenges for female PC nurses in KSA and how these might be addressed. PRISMA provides a systematic, comprehensive and clear review to reduce potential bias. We followed the five steps of an integrative review (48) to ensure rigor and structure. These inform our research strategy based on a solutions-oriented protocol (49).

The review did not require institutional ethical approval. Firstly, we designed our research questions based on search terms and insights from a previous literature review on foreign PC nurses’ employee voice in KSA (50). The study followed Whittemore and Knafl’s (48) five-step methods of problem identification, literature search, data evaluation, data analysis, and presentation.

**Figure 1:**
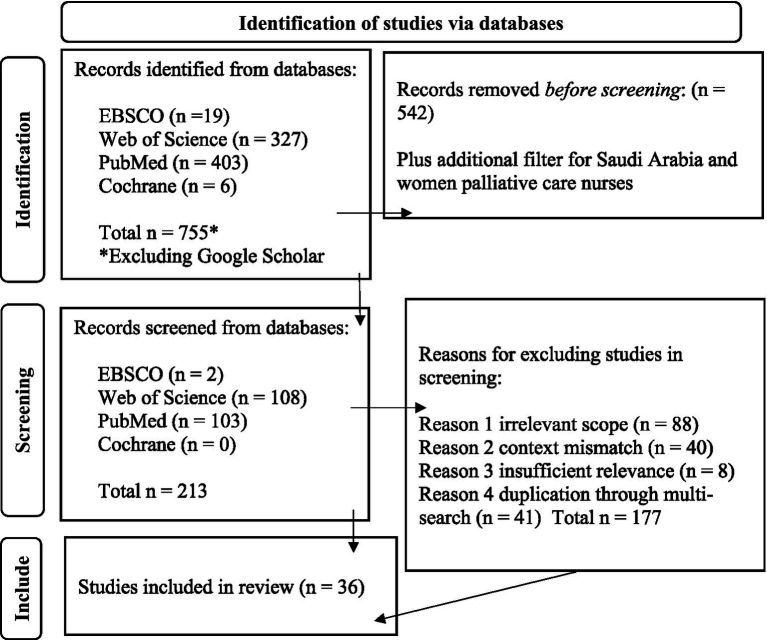
PRISMA 2020 (47) flow diagram for the literature review.

After initial scoping and to aid the literature search stage, we mobilized the PRISMA statement (47) to ensure a systematic, consistent and rigorous process in our search. We focused on established databases – Cochrane Reports, EBSCO, PubMed and Web of Science-which originally generated 755 studies. Five hundred and forty two studies were removed before screening and then 177 were removed for reasons listed in Figure 1, leaving 36 articles. We initially conducted a Google Scholar scoping search but rejected this strategy. The focus was on peer-reviewed academic literature, not grey literature. Google Scholar is unsuitable for systematic literature reviews (51). Figure 1 illustrates the three main identification processes, screening, and eligibility, consistent with established PRISMA principles. Subsequently, a thematic analysis of these articles was conducted (52, 53) to provide in-depth insights into the social determinants of health applied to female PC nurses in KSA.

The PRISMA (2020) Flow diagram^1^ was developed in line with PRISMA2020 guidelines (47).

Epistemologically, our approach is underpinned by our subjective, interpretivist philosophical stance, whereby we posit gender, sexism, and discourse as social products and discourse as co-constructed between actors within the discourse (54, 55). The positionality of the first two female scholars based in UK business schools who conducted the literature search and initial analysis focused on highlighting inequalities in the Saudi nursing workforce. The male research team member from the UK currently works in the KSA healthcare sector as a senior PC advanced clinical practitioner nurse.

## Limitations, strengths and future research avenues

This review is subject to sampling biases, focusing on evidence from KSA rather than on regional perspectives in the Middle East. Several of the studies (21, 38, 56) concentrate on Riyadh, while others are national (40). Bias may also occur in the researchers’ interpretations as they are not female PC nurses, Muslims or Saudi nationals.

Despite these limitations, the study’s strengths lie in the systematic literature search process and expertise of the three-member multidisciplinary research team, with two researchers involved in the initial search and then members checking for the credibility of publications and relevance. One team member is a highly experienced senior PC nurse currently working in KSA. All the authors have worked/carried out fieldwork in the Kingdom in healthcare. Additionally, the first author has been a trustee of a UK hospice for six years. Overall, the literature review provides valuable insights into current PC challenges in KSA with recommendations for improvements in the context of limited publications in this field.

Two researchers screened the papers initially and coded them separately. Some discussion was held between these two researchers and the advanced clinical practitioner in the team about including articles related to PC physicians. It was agreed to include these to provide context about key workforce challenges and burnout. While the authors sought to avoid bias in selecting the studies by clearly stating the selection criteria (57), we did not assess individual studies for bias as none denied that there are no challenges in KSA healthcare for nurses.

In the next section, we discuss findings that emerged from the review with implications for future PC research on nursing in KSA, in the context of Vision 2030.

## Findings from the literature review

As outlined in the PRISMA flow chart (Figure 1) after all source identification, screening and inclusion/exclusion steps were conducted, 36 articles were identified and thematically analyzed to answer the two key research questions. We sought to provide descriptions from extant literature to understand how SDOH shape female nurses’ experiences in the context of Saudi national transformation to improve SDOH within general population health.

Table 1 summarizes our key findings. The overarching argument is underscored by the established logic that population health is linked to the health of healthcare workers, as outlined by the WHO (58) report *Global strategy on human resources for health: Workforce 2030*. It supports the notion that improvements in “UHC and improvements in health care are workforce enabled” (59) (p. 860).

**Table 1:** Summary of the 36 studies reviewed.

Authors	Aims	Methods, Sample	Results	Recommendations
1. Aboshaiqah (2016) (44)	How to address nursing shortages in KSA.	Literature review 1993–2013 on cultural traditions, beliefs, nursing education and policies.	KSA depends largely on an expatriate nursing workforce. Shortages of Saudi female nationals, especially women. Cultural factors exacerbate poor image of the nursing profession.	Calls for Saudisation of the workforce, improving attracting and retaining Saudi nationals. Solutions: education and media messages, improving the workplace–teamwork, sufficient staffing, addressing some aspects of culture with greater compatibility for Saudi nationals.
2. Abudari et al. (2016) (2)	Non-Muslim nurses’ experiences of caring for Muslim palliative care patients.	Qualitative descriptive study, 10 interviews nurses medical, oncology, and oncology/PC units tertiary care hospital in KSA. Modified Stevick–Colaizzi–Keen method for data analysis.	Family matters, end-of-life practices, nurse challenges related to cultural values, religious practices. Lack of PC integration, members of interdisciplinary PC team unavailable. Nurses’ lack of cultural knowledge and formal cultural education.	Introduce cultural care nursing delivery model with Islamic teachings and practices to understand religion, culture and family’s role.
3. Alabd et al. (2024) (60)	Nurses’ working life quality in primary healthcare in Madinah City.	Descriptive cross-sectional design, on-line survey, 290 primary healthcare nurses, quality of nursing work life scale.	Half (45.9%) had a high work life quality level, 43.8% had a moderate level, and 10.3% had a low level. Key influences: job structure, work/home life, and work context subdomains. Age, income, nationality, marital status, number of children affected nurses’ work life quality.	Nurses’ quality of work life ranged from moderate to high: age, income, nationality, marital status, and number of children influenced nurses’ work life quality. Improving quality of nursing leadership can lead to enhanced patient and nurse satisfaction and quality care.
4. Alanazi et (2024) (64)	Ethical challenges in end-of-life decision making in PC nursing. To evaluate communication and decision-making strategies and identify approaches to support nurses and patients.	Systematic review using PRISMA guidelines. The risk of bias was assessed using ROBVIS-II. analyzed thematically.	22 studies. Key themes: (1) Effective communications and engaging patients in decision-making are critical but complex. (2) Nurses must balance autonomy, beneficence and relational issues. (3) Integrating PC principles enhances symptom management, aligning care with patient values. (4) Education and organizational support must equip nurses with appropriate skills and coping strategies.	It is vital to address interconnected ethical, communication and support needs. Further research is required on cultural competence training, standardized education programs and longitudinal evaluations.
5. Albejaidi and Nair (2021) (14)	Nationalization of the Saudi public and private health workforce.	Dependency on expatriate health workforce. Saudisation policy overview, underlying impact on the health workforce in public and private sectors.	Strategic government initiatives for health workforce development and challenges in replacing expatriates within Vision 2030. Low participation of females in nursing, mainly due to a poor image of the profession, and religious and cultural factors.	Adopt a health workforce development policy that integrates local culture, values and social ties. Government needs to change the mindset of citizens to take up health care jobs, particularly in nursing.
6. Alboliteeh et al. (2017) (61)	Profiling the Saudi nursing workforce. Saudisation policy to reduce reliance on the expatriate workforce and unemployment rate of Saudi national.	Cross-sectional questionnaire, 741 responses. Saudi Registered Nurses working in MOH hospitals.	An increase in the recruitment of Saudi males may reflect cultural issues such as gender specific facilities.	Saudisation program adopts a non-discriminatory approach to employment of both genders into nursing.
7. Aldekhyyel et al. (2024) (40)	Saudi women’s views on healthcare leadership during Saudi Vision 2030 which emphasizes women’s empowerment and their increased participation in the workforce, particularly in healthcare.	National cross-sectional on-line survey based on the Leadership Effectiveness Model. Women classified as “Consultants” by the Saudi Commission for Health Specialties. Descriptive statistics and content analysis. 119 Saudi women consultants participated. Physicians (85%) in the Western region, (46%) in leadership roles.	Leadership positively impacted career growth but negatively affected leisure activities. Career progression challenges: further studies (35%), work-life balance (31%). Leadership commitment to supporting women was seen as crucial (63%). Vision 2030 focus on “advancement,” “opportunities,” and “empowerment,” with evolving organizational cultures and policies is creating opportunities for women to excel in healthcare leadership positions.	National strategies, combined with workplace norm changes and supportive policies, can foster greater representation of qualified women in top healthcare leadership positions.
8. Aldossari and Calvard (2022) (29)	Political and ethical perspectives on resistance, feminism and gender in Saudi organizations. Women’s experiences of gendered segregation, under-representation and exclusion despite greater numbers of women entering Saudi workplaces.	Interview study with 58 Saudi Arabian women to explore their attitudes toward gender-segregated and mixed workplaces.	Novel conceptualization of how gender is experienced through patternings of resistance and conformity shaped by the ethicopolitical, religiopolitical and sociocultural webs of meaning in Saudi society that permeate workplaces. Saudi women’s nuanced commitments and resistances to context-relevant identity issues.	There is a need for feminist ethics and politics of gendered resistance and conformity to working practices and norms in Islamic and Middle Eastern settings.
9. Aldossari and Chaudhry (2024) (32)	Examines patriarchal culture and state policies affecting the Saudi labor market. The study explores the intersection of state-driven policies, patriarchal culture, and gender precarity.	Saudi Arabian retail sector, 26 in-depth interviews with employees and other stakeholders. Focuses on the multi-layered nature of precarity, individuals’ subjective experiences of precarity against a backdrop of structural precarity.	For Saudi men, state-driven policies exacerbate job insecurity and challenge traditional family ideology and the breadwinner model. Saudi women face socio-economic vulnerability and organizational neglect, resulting in under-reporting of sexual harassment and limited protests. This antagonistic interplay of state policies and entrenched socio-religious norms creates structural and subjective precarity in workplaces.	Highlights the complexities in addressing gender disparities, intersectionality of gender, religiosity, and power relations. Illustrates how state policies and patriarchal culture shape structural and subjective forms of precarity. Emphasizes the importance of fostering feminist consciousness amongst women to address gender inequalities.
10. Aldossari and Murphy (2024) (28)	Gendered moral rationalities in KSA. How women actively negotiating their inclusion in response to societal and organizational contexts as part of the first generation to enter the workforce.	Economic objectives in Vision 2030. Significant increase in women’s workforce participation. Relational lens on inclusion theories.	56 interviews with Saudi Arabian working women, how women negotiate tensions between labor market participation and societal gender ideals.	Applying gendered moral rationalities to capture the complexity of the interplay between women’s gender roles and work roles, identified three orientations: *traditionalists*, *pragmatists* and *trailblazers.* Need for current workplace inclusion theories to focus on ways individuals actively negotiate workplace inclusion in the broader societal context in addition to organizational efforts.
11. Al-Dossary (2022) (24)	Links between nurses’ quality of work-life, organizational loyalty and job performance in Saudi hospitals.	To analyze the relationship between quality of work-life on organizational loyalty and job performance in KSA.	Cross-sectional design, hospital nursing staff. Three questionnaires: Quality of Work Life Scale (QWLS), Organizational Commitment Questionnaire (OCQ), and Individual Work Performance Questionnaire (IWPQ). Statistical analysis of 209 responses.	Nurse managers reported good quality of life and high loyalty towards their employers as well as good job performance levels. However, staff nurses reflected poor quality of work-life, organizational loyalty, and job performance. Training and development had strong positive correlation with continuance commitment. Job satisfaction and job security held strong positive correlation with task performance and contextual performance. Poor quality of work-life can negatively impact nurses’ job performance and organizational loyalty.
12. Al-Dossary (2018) (71)	The future of the nursing profession and Saudi Vision 2030.	Challenges remain in building and sustaining a Saudi nursing profession and workforce to support population and economic growth.	Review of 19 studies published 2001–17 on nursing shortages, under-developed nursing education and unclear scope of practice.	Effective strategies must be implemented to advance the nursing profession and to improve healthcare delivery. Nursing policymakers urgently need to improve nursing care in Saudi Arabia by addressing the nursing shortage, generating strategies to improve nursing education and establishing scope of practice guidelines.
13. Alhatim and AlShehery (2024) (21)	Saudi physicians’ views on PC challenges in KSA.	Explores perceptions of physicians in Riyadh of PC challenges.	Cross-sectional survey, electronic questionnaire distributed among physicians involved in palliative care, SPSS analysis. Sample 20–40 years old (48.48%, *n* = 96). Male physicians accounted for 64.65% (*n* = 128). Specialties: critical care (15.66%, *n* = 31) and radiation oncology (16.67%, *n* = 33).	Strategies needed: creating triage systems, using telemedicine, advanced care planning. Significant barriers: limited services, ethical dilemmas, lack of telemedicine facilities requiring ethical support for healthcare providers, integration of telemedicine, continuous education, improved access to multidisciplinary care teams for comprehensive patient support.
14. AlKhalifah et al. (2024) (11)	To estimate the rate and associated factors of burnout and its dimensions among healthcare workers of the Palliative Care Department in King Fahad Medical City, Riyadh.	Observational cross-sectional study. Pre-designed, valid self-administrated questionnaire on sociodemographic and work-related data and Maslach burnout inventory (MBI) to assess the overall burnout and its three subscales: emotional exhaustion (EE), depersonalization (DP), and personal accomplishment (PA).	Prevalence of high EE (49.3%), those of high DP and low PA were 62 and 63.4%. Overall prevalence of burnout among PC health workers 23.9%. Divorced/widowed participants were at significantly higher risk of burnout. Those with long annual vacations (>4 weeks) were at lower risk of burnout.	Burnout is prevalent amongst healthcare workers working in PC in Riyadh. Decision makers should take care of the working environment, annual vacation length, and divorced/widowed workers to reduce burdens causing burnout.
15. Alluhidan et al. (2020) (39)	Nursing challenges and policy opportunities in KSA.	Case study summarizes Saudi Ministry of Health and Saudi Health Council’s evaluation of current challenges facing the nursing profession.	From a nursing human resources for health (HRH) perspective: challenges of low nursing school capacity, high employment of expatriates, labor market fragmentation, shortage of nurses in rural areas, uneven quality, and gender challenges. Need for policy interventions to support the transformation of nursing into a profession for efficient, high-quality healthcare.	MOH to collaborate with partners across the healthcare system, particularly the private sector. Human resources planning sector-wide and nursing leadership strengthened at all levels. Improve pipeline of nurses from middle and high school to nursing school; diverse career path including postgraduate education. Retention: making nursing practice more attractive and family friendly. Reducing shift length, redesigning nursing teams with more allied health workers, and temporary staffing to balance workload. Modernize existing nurse postgraduate education, open new postgraduate nursing programs, create new positions and career paths, e.g., telenursing, informatics, quality assurance. Rural pipelines with incentives and increased compensation packages for under-served areas.
16. Almansour et al. (2020) (26)	Nurses’ nationality and job satisfaction in Saudi hospitals.	To examine whether there is an association between nationality and nurse job satisfaction.	Cross-sectional survey, 743 nurses from three major government hospitals. Job satisfaction measured using McCloskey/Mueller Satisfaction Scale. May 2014–February 2015.	Expatriate nurses had overall lower job satisfaction than Saudi nurses. Expatriates were less satisfied about extrinsic rewards and family–work balance. Saudi nurses were less satisfied about their professional opportunities, praise and recognition, and co-worker relationships. Solutions: effective induction programs that help newly employed nurses – migrant and local – to understand their jobs, roles and responsibilities clearly. Policy makers should collect evidence to modify the reward system to ensure fairness and equality for all.
17. Almobarak (2024) (1)	Expanding PC access in KSA.	To assess past trends in adult PC needs in KSA.	Population-based secondary data analysis using two PC needs estimation methods: the direct or fixed estimation method by Gómez-Batiste and the maximum or maximal method by Murtagh and Rosenwax. Estimated PC needs were stratified by gender. By 2022, the number of people requiring palliative care skyrocketed to 79,725 (fixed method) and 101,623 (maximal method).	An upward trend in PC needs is evident through the estimation techniques. The demand for PC in KSA substantially rose in the observed years. The gap between PC needs and supply must be addressed with advanced integration of palliative care services in the national healthcare system.
18. Almujadidi et al. (2022) (38)	The case for a multidisciplinary approach to exploring SDOH in Saudi primary care.	Identified barriers and enablers for addressing SDH in clinical settings in KSA, considered the influence of local cultural and social norms to improve care and support for marginalized and under-served patients.	Qualitative study, individual in-depth interviews 17 primary health care physicians purposefully selected. Focus group with four social workers, all recruited from King Khalid University Hospital in Riyadh. Thematic analysis using a deductive-inductive approach. Financial burdens, challenges in familial dynamics, mental health issues and aging population difficulties were common social problems. Action on SDH in primary care was hindered by (1) lack of physician knowledge or training; (2) organizational barriers including time constraints, patient referral/follow up; (3) patient cultural norms and (4) lack of awareness of physicians’ roles in managing SDH.	Enablers for more socially accountable care: (1) more education and training on addressing SDH in clinical care; (2) organizational innovations to streamline identification of SDH during patient encounters (e.g., case finding questionnaire completed in waiting room); (3) better interprofessional co-ordination and clarification of roles; (4) identifying opportunities for broader advocacy to improve living conditions for marginalized groups. Need for (i) training physicians to help patients in navigating social challenges; (ii) improving clinical/administrative interprofessional teams; (iii) mobilizing local communities in addressing social challenges; and (iv) advocating for preventative intersectoral healthcare actions.
19. Almulla and Hassouneh (2022) (4)	Integrative review of home-based PC and home health care in KSA. It synthesizes and analyses literature relevant to home-based PC on nursing and home health care (HHC).	Only 14% of PC patients worldwide receive home-based PC. Many people prefer to receive care and die at home. Nurses are critical to successful delivery of home-based PC.	24 studies published during 2005–21. Lack of nurses’ knowledge and awareness of PC and the under-development of HHC in KSA have contributed to under-used services.	Nurses are vital to the functioning of interdisciplinary teams and effective interfacing with patients, caregivers, and families. Education and training of nurses in KSA are essential for promoting access to PC and HHC and the development of home-based PC.
20. Alqahtany et al. (2025) (20)	Demographics and distribution of PC physicians and future challenges in KSA.	Descriptive geolocation study in 2024 using Saudi Commission for Health Specialties data and the 2022 Saudi Census. Analysis of the number, distribution, and characteristics of palliative care physicians across 13 cities and 26 hospitals in KSA. Comprehensive literature review to contextualize these findings.	110 PC physicians in KSA, 84.5% Saudi nationals. The physician-to-population ratio was 1: 292,500 with significant regional disparities. Only 9% of Ministry of Health hospitals had PC services. Key challenges: workforce shortages, uneven distribution of services, limited integration into primary care.	Recommendations: expanding medical education and training programs, improving service distribution in under-served regions, integrating PC into primary health care.
21. Alreshidi et al. (2021) (30)	Turnover of foreign nurses in KSA which influences costs, healthcare accessibility and quality, financial proficiency of healthcare systems.	Quantitative-based cross-sectional descriptive study, survey for statistical inferences about foreign nurses’ turnover in the Ministry of Health (MOH) hospitals.	Factors influencing turnover were categorized into nine dimensions: professional growth and development, leadership style, management, wage and benefits, workload, interpersonal relationship, housing facilities and services, hospital facilities and intent to stay and turn-over intention, of which the professional growth and development had the highest mean agreement scores, whereas housing and hospital facilities showed the lowest mean scores.	Wage benefits and workload factors were the most significant causes of expatriate nursing turnover, closely followed by inadequate housing and hospital facilities.
22. Alrimali et al. (2024) (65)	Evaluated the education, practice, and perceived competence of adult ICU (intensive care unit) nurses in KSA regarding palliative and end-of-life (PEOL) care.	Cross-sectional design. 142 ICU nurses completed the survey, recruited from five public hospitals and one specialized center in Hail. Data collected in September 2023 using the PEOL Care Index, Likert scale in Arabic and English. Statistical analysis.	81% of the nurses had experience caring for dying patients but only 30.3% had received in-service PEOL care training. Those with this training demonstrated significantly higher scores in education, clinical practice, and perceived competence. In-service training positively correlated with these metrics. In-service training, job satisfaction, and communication authority are strong influencers.	The study highlighted the proficiency of ICU nurses in PEOL care, emphasizing that in-service training, job satisfaction, and the authority to communicate effectively with patients and their families significantly improved clinical practice and nurses’ competence in PEOL care. In PEOL care, especially within ICUs, nurses’ unique skills are critical, yet their expertise remains under-explored, particularly in KSA.
23. Alruwaili et al. (2024) (67)	Compassion fatigue in palliative care, nurses’ wellbeing and quality of care.	Examines the experiences of geriatric nurses in PC. It aims to understand how these experiences influence their wellbeing and the quality of care.	Conducted in the Alahsa region. Qualitative methods, 12 in-depth interviews with geriatric nurses. Thematic approach, enriched by iterative reflections within a multi-disciplinary research team.	Main themes: (1) deep emotional connections between nurses and their patients; (2) compassion fatigue, high patient mortality, communication hurdles; (3) impact of these challenges on the quality of care, e.g., diminished empathy; (4) nurses’ coping strategies, self-care practices and continuous education.
24. Alsadaan et al. (2021) (42)	An integrative review of nursing profession challenges in KSA.	Literature review 2000–20.	Inadequate numbers of Saudi nurses, increased recruitment of expatriate nurses. Challenges in retention, lack of competency in English and Arabic, lack of understanding Arabic culture, insufficient experience, high workload and job dissatisfaction. Expatriate nurses prefer to work in developed countries. There is a need to improve the image and culture of nursing to recruit more Saudi nurses – issues in education and the working environment.	Need to improve recruitment processes for expatriate nurses, thorough education programs to improve knowledge and skills to equip them to work and stay in KSA. Organizational changes to increase job satisfaction and retention of nurses generally. Healthcare in Saudi Arabia also needs leaders to manage nursing workforce challenges efficiently.
25. Alshammary et al. (2024) (9)	Update on PC provision in KSA linked to Vision 2030 reforms in improving the quality of life for terminally ill patients and their families by improving the availability and quality of PC services.	Cross-sectional survey-based research conducted at a MoH health care facility to assess the accessibility and quality of PC services. Survey collected quantitative and qualitative data from PC managers in Saudi Arabia. Retrospective analysis of annual death records determined the demand for PC.	Increased number of PC units, community home care services, outpatient services, and consultations. Challenges persist related to geographical distribution, resource allocation, and the availability of pain medications, especially opioids. The study highlights the substantial need for PC for both cancer and non-cancer patients, emphasizing the importance of growing these services.	To further improve PC, policymakers and stakeholders must prioritize resource allocation, the health care workforce, and access to pain medications. These efforts will address the growing demand for PC and benefit terminally ill patients and their families in KSA.
26. Alsolami et al. (2023) (22)	Knowledge and attitudes towards PC and end-of-life decision-making in KSA, including healthcare providers and non-clinicians.	To assess knowledge and attitudes regarding palliative care and end-of-life decision-making in Eastern and Central provinces among locals.	Cross-sectional survey-based research design. Purposive sampling using social media. Data included demographic information, PC knowledge, attitudes toward PC, and cultural influences on end-of-life decisions. 710 participants completed the survey, with a balanced gender distribution, predominantly aged 25–54, over half were healthcare providers, many had over 15 years’ healthcare experience.	A substantial proportion had received formal PC training and were personally involved in end-of-life decisions. Most demonstrated a good understanding of PC. There were knowledge gaps, especially related to its timing. Generally, participants felt at ease discussing end-of-life care and believed in PC’s effectiveness. Need for targeted education to rectify misperceptions, particularly concerning the initiation timing of palliative care, and the value of culturally sensitive healthcare discussions and provider training.
27. Al-Yami et al. (2018) (66)	Nursing staff’s leadership style and organizational commitment in KSA.	Examine link between nurse managers’ leadership styles, and nurses’ organizational commitment. Effective leadership is influential in staff retention. Recruiting and maintaining nurses is an increasing problem in KSA.	Multifactor Leadership Questionnaire and the Organizational Commitment Questionnaire distributed to 219 nurses and nurse managers in two hospitals.	Transformational leadership was the most dominant leadership style which contributed to organizational commitment. Perceptions of both transformational and transactional leadership styles increased with age for nurse managers and nursing staff. Introducing the Full Range of Leadership model to the Saudi nursing work force could help to prepare Saudi nurses for positions as nurse managers and leaders. Transformational leaders could influence and induce positive changes in nursing.
28. Batayneh et al. (2019) (62)	Foreign nurses’ burnout in KSA. Reliance on overseas nurses unique working environment, most nurses are working outside their home countries.	Relationship between workplace stress, job satisfaction, intention-to-leave and the development of burnout among multinational nurses in KSA.	KFMC Riyadh has a 3,000 multinational nursing workforce. Correlational, cross-sectional study, 1 August-30 December 2016. 224 nurses completed survey.	Workplace stress and nurses’ intention-to-leave are positively correlated with the development of burnout symptoms. Job satisfaction levels had a negative correlation with the development of burnout symptoms. Overall, nurses’ demographics had no significant effect on the development of burnout. Multinational nurses in KSA suffer from work-related stress and burnout that can translate into high turnover, which in turn can be detrimental to national health organizations.
29. Callaghan et al. (2024) (56)	Family caregivers’ satisfaction and their views about the burden of patients’ symptoms in a tertiary palliative care service (PCS).	In a tertiary care center in Kingdom of Saudi Arabia (KSA).	Cross-sectional design, convenience sample 264 family care givers known to a tertiary care center, May–September 2023. Family Satisfaction with End-of-Life Care Scale (FAMCARE-2) and Arabic Questionnaire for Symptom Assessment. 94% response rate. Male participants (*n* = 55.7%), mainly aged 30–50 years. Approximately half were receiving disease-modifying treatments, 38.3% had a Do-Not-Attempt-Resuscitation order.	High satisfaction with how the services respected dignity. Less satisfied with ‘the practical assistance provided by the PCS.’ Satisfaction was higher in the outpatient setting (inpatients are often more unstable and symptomatic). The most severe symptom reported by FCs was ‘tiredness’, followed by ‘pain’.
30. Chowdhury et al. (2021) (15)	Saudi health transformation and new model of care.	Describes the new Model of Care (MOC) within Vision 2030. Six systems of care (SOC) – keeping well, planned procedure, women and children, urgent problems, chronic conditions, and the last phase of life.	KSA needs to modernize the health care system to reach Vision 2030 goals. The new MOC describes a total of 42 interventions, with 27 split across the six SOC and the 15 cut across the multiple SOCs.	Implementation of all MOC interventions will streamline the Saudi health care system to embrace the Kingdom’s “vision 2030.”
31. Davies et al. (2024) (50)	Voice and intersectional inequalities of migrant PC nurses in KSA. Value of culture care theory.	Narrative literature review of 31 articles, examines intersectional employee voice inequalities to develop a multilevel conceptual framework using a culture care model.	Need to improve transcultural communications and reduce voice inequalities that influence migrant employee wellbeing and intentions to quit which result from cultural incongruities.	Further research is needed on the impact of national cultural differences and intersectional inequalities on employee voice. Policy ambitions for advancing healthcare cannot be realized while discriminatory practices based on conservative attitudes which stifle migrant worker voices.
32. Elmorshedy et al. (2020) (68)	How the nursing profession is perceived in KSA.	To explore the level of community awareness and public image of the nursing profession in Saudi Arabia.	Cross-sectional study, convenience sample 502 adults including106 males and 396 females, mean age 22.93 ± 6.76 years. Data collected socio-cultural data, gender preference in receiving nursing care, awareness, perceived socio-cultural barriers to pursue a nursing career.	Only 32.5% preferred to receive nursing care from Saudis. 71.5% of participants would be ashamed of having a nurse in their family. Socio-cultural barriers to pursuing a nursing career: gender-mixed working environment (35.9%), delayed marriage of female nurses (20.3%), negative effect of nursing profession on social life (64.5%). Half of the sample had poor awareness. Negative stereotype of the nursing profession among Saudis.
33. Ghazwani (2022) (23)	Burnout amongst PC clinicians in KSA.	Assess the prevalence and risk factors associated with burnout among PC clinicians in KSA.	Cross-sectional study in 2018, 44 PC physicians (26 males and 18 females). Maslach Burnout Inventory’s (MBI) 22-point scale questionnaire to assess emotional exhaustion, depersonalization, and reduced personal accomplishment. Pearson correlation and binary logistic analysis were performed using SPSS to find out factors influencing burnout considering *p-*value of <0.05 as significant.	Eight participants (18.2%) experienced emotional exhaustion and 11 (25%) experienced depersonalization and detachment, and reduced personal accomplishment, each. Job title and availability of administration supporting health care staff, and pain relief medications have shown significant impact on burnout levels. Overall low prevalence of burnout.
34. Habib (2022) (13)	Significant gender gap in Saudi citizens accessing healthcare services.	To assess gender gaps in accessing primary healthcare among Saudi citizens in urban and rural areas.	Survey of 529 respondents, male and female citizens of Makkah city.	Statistics confirm significant differences in healthcare access by gender. The availability of services, as well as the barriers to access, must be evaluated in the context of varied groups in society’s differing perspectives, health requirements, and material and cultural surroundings.
35. Maawadh et al. (2024). (63)	What influences female health sector employees’ satisfaction in KSA?	Assesses the satisfaction of Saudi women working in the health sector, factors in the work environment, factors that enable them to gain opportunities and benefits and make decisions.	Cross-sectional study, closed-ended surveys 261 Saudi women working in the healthcare sector. The findings showed a significant association between female workers in health sector satisfaction with factors related to the workplace environment, training, and development, and their involvement in decision-making.	Most women felt empowered when they received equitable tasks and were able to reach managerial-level positions in their organizations. It is important to create a positive work environment with opportunities which enhance women workers’ satisfaction.
36. Tayeb et al. (2010) (3)	Perspectives on what makes a “good death” for Muslim patients and healthcare givers.	Review of The Future of Health and Care of Older People (TFHCOP) report good death perception to determine its validity for Muslim patients and health care providers.	284 Muslims, both genders, different nationalities and careers. Questionnaire based on 12 principles of the TFHCOP good death definition, followed by face-to-face interviews. Descriptive statistics to analyze questionnaire responses. For new themes, a grounded theory approach was used with a “constant comparisons” method.	On average, each participant agreed on eight principles in the questionnaire. Top priorities: dignity, privacy, spiritual and emotional support, access to hospice care, ability to issue advance directives, and to have time to say goodbye. Three main domains: faith and belief; principles related to self-esteem and person’s image to friends and family; satisfaction about family security after the patient’s death. Professional role distinctions were more pronounced than gender or nationality differences. Many Muslim patients and health care providers did not recognize as important several aspects of a “good death” as perceived by Western communities.

Four themes emerged in an analysis of the literature related to equity, ability, compassionate support, and meaning in the workplace.

### Equity

The literature linked to social determinants of health for female nurses related to issues of equity in the workplace related to unfairness, discrimination, heavy workloads, and a lack of respect for nurses in the general population. These factors exacerbate the shortage of Saudi female nurses. Cultural factors explain the poor image of the nursing profession (44). There is discrimination against Saudi female nurses due to cultural perceptions and socio-economic factors. Alabd et al. (60) identified unfair pay and negative and inequitable treatment of nurses based on their nationality and marital status impact nurses’ work-life quality. Work-life balance and job structure affect nurses differently based on their nationality and socio-economic status. Albejaidi and Nair (14) explored gender disparities. They found that the low participation of Saudi women in nursing results from cultural and religious barriers, as well as the poor image of the profession. There is discrimination against female workers due to cultural norms and the undervaluing of nursing as a career.

Alboliteeh et al. (61) noted that the Saudisation policy to increase the number of local nurses is impacted by cultural gender norms, leading to more male than female Saudi nurses being recruited. In the Kingdom, Aldossari and Calvard (29) examined gender-based segregation and the underrepresentation and exclusion of women in KSA workplaces. Their study highlighted structural barriers and socio-cultural resistance. The authors argue that Saudi women experience systemic discrimination despite their increasing participation in the workforce. In addition, Aldossari and Chaudhry (32) discuss patriarchal culture, gender precarity and job insecurity with cases of sexual harassment under-reported and a lack of workplace protection for women. They highlight Saudi women’s socio-economic vulnerability and organizational neglect.

AlKhalifah et al. (11) examined burnout among healthcare workers in PC, including factors such as emotional exhaustion, depersonalization, and personal accomplishment. Batayneh et al. (62) also demonstrated that foreign nurses’ burnout is due to inequalities in how they are treated in the workplace, high stress levels and job dissatisfaction, which results in high turnover rates in KSA hospitals. Insights into how burnout amongst PC clinicians in KSA can be reduced by administrative support (23) could also be applied to nurses. PC staff expressed concerns about work overload, emotional strain, and psychological safety concerns that lead to inequitable working conditions. Almansour et al. (26) found that job satisfaction among nurses is based on nationality and characterized by socio-economic discrimination and pay unfairness among expatriate nurses, especially from India and the Philippines. The turnover of foreign nurses is due to high workloads, inequities in wage benefits, inadequate housing, and poor hospital facilities (30). Clearly, work overload, financial stress, and insufficient living conditions are key equity issues affecting foreign nurses. Moreover, Davies et al. (50) noted intersectional inequalities (based on age, gender, nationality, experience, education, and occupational status, for example) among migrant PC nurses in terms of voice inequalities and cultural discrimination. Maawadh et al.’s (63) research on Saudi female healthcare workers’ dissatisfaction with the workplace environment showed their concerns about a lack of involvement in decision-making. Inequities discussed in the literature related to female nurses reflects a significant gender gap in the general population and inequalities in women accessing healthcare services in KSA (13).

### Ability

A focus on the need for better knowledge, skills, education, competencies, and training also characterizes the literature on nursing in KSA. Abudari et al. (2) highlighted non-Muslim nurses’ challenges in understanding Islamic cultural and religious practices when providing PC. For example, there is a lack of cultural knowledge and formal education about end-of-life Muslim patients’ expectations. Aboshaiqah (44) highlighted gaps in nursing education and training gaps as barriers to attracting Saudi nurses to the profession. There are calls for enhanced education and media strategies to improve the image of nursing and to increase recruitment (42). Alanazi et al. (64) explored ethical challenges in end-of-life care and the need for training in communication, decision-making, and PC principles to support nurses. Cultural competency training and standardized education can improve nurses’ coping strategies. Albejaidi and Nair (14) proposed educational and training reforms to encourage Saudi citizens to pursue healthcare roles, including nursing.

There is a need for workforce development policies to integrate cultural and social values. Al-Dossary (24) discussed nursing shortages, inadequate education, and unclear scope of practice that require better education and training reforms in nursing education and clearer definitions of practice guidelines to enhance workforce capabilities. Alhatim and AlShehery (21) see potential solutions to a lack of knowledge and understanding of PC as a greater understanding of telemedicine, advanced care planning, and ethical challenges in PC. They advocate continuous education and training, integrating telemedicine and multidisciplinary care to improve PC services. Alluhidan et al. (39) reviewed nursing challenges and policy opportunities in KSA. A key theme is addressing nursing skill gaps, training needs, and policy interventions to strengthen workforce capability. Alqahtany et al. (20) analyzed the distribution and demographics of PC physicians and identified challenges in workforce planning and medical education to address workforce shortages and future skill development needs in PC. Alrimali et al. (65) considered ICU nurses’ competence in palliative and end-of-life (PEOL) care, showing training gaps and the impact of education. They demonstrated how in-service training improves competence and job satisfaction among ICU nurses. In another study, Alsadaan et al. (42) pointed to language barriers, cultural understanding, education deficits, and competency issues among expatriate nurses as key challenges. Al-Yami et al. (66) looked at nursing leadership styles and organizational commitment. They emphasized the role of transformational leadership in improving nursing retention and competence, linking leadership development to workforce capability and professional growth.

Furthermore, Alsolami et al. (22) emphasized the lack of general knowledge and negative attitudes towards PC among healthcare providers and the public. They identified gaps in PC education and the need to change misconceptions about the specialism with calls for targeted education initiatives to improve public and professional understanding of PC.

In another study, Alrimali et al. (65) explored ICU nurses’ education, practices, and perceived competencies in PEOL care, again stressing the need for in-service training. Alrimali et al. (65) emphasized the value of empowering PC nurses in KSA through ongoing specialized training to improve their competencies, confidence and scope for decision-making for better patient care, communications with patients and families and job satisfaction. Alsadaan et al. (42) reported that challenges in the Saudi nursing profession for expatriates include language competency, cultural understanding, and education. Alsolami et al. (22) also mention the need to improve knowledge and attitudes towards PC and end-of-life decision-making through education.

Interestingly, Tayeb et al. (3) contributed to perceptions of a “good death” among Muslim patients and healthcare providers with a need for a better cultural understanding of how views differ in the East from the West. Al-Yami et al. (66) considered links between nurse managers’ transformational leadership styles and organizational commitment in nursing staff. This points to developing leadership competencies in the nursing specialism.

### Compassionate support

Literature also indicated the importance of changing organizational workforce policies to enable inclusive cultures, positive team relationships, and compassionate line management support. For instance, Alabd et al. (60) examined work-life quality and the role of leadership in improving nurses’ experiences in primary healthcare settings. They emphasized the importance of leadership support and job structure in ensuring job satisfaction and quality of care for patients and their families. Additionally, Aldekhyyel et al. (40) stressed the importance of women’s leadership in healthcare and the impact of leadership roles on work-life balance. Aboshaiqah (44) recommended improving teamwork and staffing levels to create more supportive working environments. This requires ensuring sufficient staffing and better team collaboration to reduce stress and improve retention. From a systems viewpoint, Alanazi et al. (64) noted the need for workplace policies and support systems to help nurses navigate ethical and communication challenges in PC, such as ethical decision-making. Al-Dossary’s (24) work on nurses’ quality of work-life, loyalty, and job performance illustrated the negative impacts of poor working conditions on job satisfaction. They show that job security and satisfaction strongly correlate with nurses’ performance and loyalty. Alhatim and AlShehery (21) identified limited services and lack of ethical support for providers as barriers to PC delivery and recommended the need for better team coordination with improved access to multidisciplinary teams for patient-centered care. To address these challenges, Almujadidi et al. (38) advocated a multidisciplinary approach to social determinants of healthy workplaces. They emphasized team-based care for marginalized patients through collaborative care models and social support in improving PC delivery. In shifting from acute to community care settings, Almulla and Hassouneh (4) highlighted the value of home-based PC and home health care where nurses are critical in delivering care outside hospitals. As demand for community and home-based PC care has grown since the COVID-19 pandemic, nurses need specific support in developing confidence and competence to deliver home-based PC and integrate home health services into the broader healthcare system.

Of course, compassion fatigue among PC nurses requires capabilities to explore emotional connections, avoid burnout, and develop coping strategies. Alruwaili et al. (67) underscored the need for emotional support and workplace wellbeing initiatives. Recently, Alshammary et al.’s (9) update on PC provision linked to Vision 2030 links patient accessibility, workforce distribution, and resource allocation. The authors emphasize policy-driven workforce support and system-wide improvements in PC delivery. On a practical level, to deal with compassion fatigue in PC and emotional burdens, Alruwaili et al. (67) talk about the necessity of self-care strategies and teamwork. In an interesting study by senior expatriate nurses on the front line, Callaghan et al.’s (56) insights into family caregivers’ satisfaction with PC services in KSA drew attention to emotional and practical support that families value, which in turn shed light on nurses’ emotional wellbeing in the context of Saudi healthcare transformation and the new model of care in Vision 2030 (15).

### Meaningful work

The fourth theme, which emerged from a thematic analysis of the literature, concerns nurses’ motivation, purpose, career opportunities, and public perceptions of nursing, which we labelled “meaningful work”.

Abudari et al. (2) argued that understanding Islamic teachings could enhance the sense of purpose and meaning for nurses who provide PC care for Muslim patients. Alreshidi et al. (30) suggested that the turnover of foreign nurses in KSA could be reduced with professional growth and development opportunities. Expatriate nurses are more likely to be motivated if they at least understand the nuances of a cultural care nursing model aligned with Islamic values.

Aldekhyyel et al. (40) examined Saudi women’s leadership experiences and opportunities for career growth in healthcare under Vision 2030, which promotes advancement and empowerment despite work-life balance challenges for healthcare leaders. However, Aldossari and Murphy (28) explored how Saudi women negotiate their workplace inclusion, categorizing them as traditionalists, pragmatists, or trailblazers based on women’s career aspirations and identity negotiations within workplace structures (50).

Elmorshedy et al. (68) commented on the public’s perception of the nursing profession in KSA with concerns about negative stereotypes and cultural barriers. Alsadaan et al. (42) also emphasized the need to improve the image and culture of nursing to attract Saudi professionals. When considering the future of nursing in KSA and Vision 2030, Almobarak (1) assessed trends in adult PC needs and the importance of improving accessibility, awareness, and public understanding to facilitate cultural understanding. Alshammary et al. (9) emphasized the importance of PC for both cancer and non-cancer patients, with growth in PC services being seen as making a meaningful contribution to national healthcare improvements. PC provision is critical for improving the quality of life for patients with life-limiting illnesses and their families. Aboshaiqah (44) highlighted the poor public perception of nursing in KSA and the need for media campaigns to change societal attitudes so that nursing is promoted nursing as a respectable career to attract Saudi nationals. Al-Dossary (24) also recommended improving the public’s views about nursing to reduce nursing shortages and attract more Saudis into the profession. Alsolami et al. (22) discussed public and professional attitudes towards PC, highlighting cultural influences on end-of-life decision-making. They argued for addressing cultural and social dimensions of PC awareness and acceptance. Albejaidi and Nair (14) reiterated the need to change public perceptions of nursing to encourage more Saudis, particularly women, to join the profession. They advocated a cultural shift in how nursing is perceived to improve recruitment.

## Discussion

The narrative literature review (69, 70) indicates how gender, foreignness and other social determinants amongst the Saudi nursing workforce affect their wellbeing and turnover intentions. Female nurses appear to be in social categories that perpetuate behaviors that may disempower them and patients, frustrating national public health transformation by not addressing SDOH within the wider population. Alreshidi et al. (30) found that a lack of professional growth and development was the main reason for the turnover of foreign nurses in KSA. On the other hand, Al-Yami et al. (15) reported that transformational leadership enhanced nurses’ commitment, especially for older nurses.

Table 2 summarizes SDOH indicated in the literature review that affects nurses’ working conditions. They point to the scope for improving expatriate nurses’ pay (26, 30) job security, (61, 71), and comfort in understanding social norms in the Kingdom (37) rather than using KSA as a stepping stone to work in a developed country (42). The relatively low socioeconomic status of the nursing profession is affecting individuals’ self-esteem and job satisfaction (42) which are diminished by burnout (62) and overwork (42).

**Table 2:** Summary of social determinants of health applied to foreign female nurses in the Kingdom of Saudi Arabia.

Social determinants of health	Foreign female nurses in KSA
1. Living conditions	Inadequate housing (30)
2. Economic policies and systems	Labor market fragmentation, high employment of expatriates (39)
3. Employment precarity	Saudisation (*nitaqat*) – replacing expatriates with Saudi workers (61)
4. Social norms	Nurses’ cultural challenges, preference to work in developed countries (42)
5. Social policies	Since 2018, women drive and wearing a hijab (a head covering), or abaya (loose robe) is no longer compulsory. The Saudi government emphasizes female empowerment through education and employment but “these policies rarely account for the local realities faced and experienced by Saudi women” (37)
6. Socioeconomic position	Low status of nursing (42) lack of awareness of nursing as a profession (68)
7. Access to affordable health services of decent quality	Overseas nurses stressed and burned out (62)
8. Education	Low nursing school capacity (39) Nurses’ English/Arabic competency, insufficient experience (42). In 2023, Saudi nurses with only diplomas completed after three years became nursing technicians (86)
9. Income and social protection	Indian and Filipina nurses are paid less (42).
10. Social inclusion	Gender challenges (39)
11. Job insecurity and working life conditions	Long shifts and working weeks, lack of flexible and part-time working (71). High workloads (42). Job dissatisfaction, attrition, prefer to work in developed countries (42)

Traditional perspectives overlook overseas female nurses’ status as skilled migrants (72). This reinforces disempowerment within organizational spaces and careers. Our insights gained from the review advance our understanding of how sociocultural intersections within organizational structures and interpersonal dynamics constrain migrant female nurses by mirroring social determinants of health in the wider population. Additionally, the article offers a broader understanding of gender as a social category and how it intersects with foreignness in ways that enable us to re-imagine policy-practice gapsas KSA seeks to increase female workforce participation and non-oil revenue within Vision 2030 (73).

A notable dearth of literature surrounds PC nursing practices related to care’s cultural aspects and dynamics (4). We argue here that the sociocultural and power dynamics between PC patients (and their families) and migrant nurses are largely neglected. Nonetheless, a growing body of literature clarifies the increasing PC requirements of the Kingdom’s ageing population when current palliative provisions are lagging (1). However, the launch of the Kingdom’s national PC program, as part of Vision 2030, is expanding access (1, 22). Yet there is a dearth of literature on developing policies to increase, support, and develop PC nurses in KSA.

The low social status of nursing in the Kingdom (42) is underpinned by a lack of awareness of and respect for nursing as a profession (68). Foreign nurses feel that they are in precarious positions (39), where they are stressed and burned out (23, 62) because of staffing shortages. Migrant PC nurses’ voices are stifled in the workplace (50) as a result of a lack of engagement from line managers. There is overt pay discrimination based on whether a nurse has a passport from an LMIC (low-and-middle income country) irrespective of high competencies, which means that Indian and Filipina nurses are paid the least (26). More difficult working conditions for migrant nurses than for locals have an adverse effect on their job satisfaction (26). In most organizational contexts, a strong correlation exists between job satisfaction and turnover. Therefore, it is important to improve equity for expatriate nurses in KSA (42). Tensions between increasing demands for specialization in PC, labor market fragmentation, and the disproportionately high employment of expatriates (39) contribute to a context where migrant nurses find themselves in inadequate housing (30). High workloads (42), poor working life conditions, such as long shifts and working weeks, and a lack of flexible and part-time work (71) exacerbate inequalities.

Geographically, there are also clear differences between urban and rural areas, with a shortage of nurses in rural areas. There is a clustering of Saudi national nurses in urban areas (39) and cities such as Riyadh and Jeddah. This demonstrates variations in access to nursing care, the quality of care, and gender challenges amongst the rural workforce, where expatriate nurses may prefer not to work because of language and other cultural and practical challenges. Further, whilst developments are occurring regarding community-based and home-care provisions, healthcare at home services remain inadequate (15).

The national policy context of Saudisation to replace expatriates with Saudi workers (61) exacerbates precarity for expatriate female nurses in the Kingdom. There have been on-going improvements in increasing the number of Saudi national nurses (42, 44). However, the overall demand for nurses continues to outstrip supply in the KSA. This undermines effectiveness and efficiency in delivering adequate health services (74) which constrains ambitions for high-quality public health delivery. There are inevitably adverse effects amongst a stressed foreign nursing workforce where workers from developing countries represent the backbone of healthcare delivery.

In summary, unfair and discriminatory working conditions add to PC nurses’ stress and high levels of turnover in KSA. A lack of knowledge, competence and confidence in PC nursing is undermining workforce wellbeing and professional development and the use of digital health technology. This serve remote and marginalized communities are under-served, which in turn limits equity in delivering healthcare services in the context of nursing shortages. Inadequately trained healthcare professionals and weak public understanding of the benefits of PC nursing must also be addressed more widely, possibly in TV soap operas and social media campaigns. Future research might draw on the work of Shuck et al. (75) on work determinants of health (WDOH), organizational conditions that influence employees’ health in employment related to the physical and social environment, capacity, stress, and the meaning of work.

## Case study: community and telehealth palliative care

One interesting example of community PC provision, which includes pain management, symptom control and end-of-life care, is King Faisal Home Health Care Riyadh. HHC enables patients to be treated in the comfort of their own homes. Home Health Care HHC started in 1991 at King Faisal Specialist Hospital & Research Centre. It is a non-profit tertiary healthcare institution headquartered in Riyadh. It offers primary and specialized inpatient and outpatient medical care and participates in clinical and research studies. HHC palliative nursing care is based on a multidisciplinary approach with specialized medical care for HHC patients with serious illnesses. HHC relieves patients from symptoms, pain, and physical and mental stress to improve the quality of life for patients and their families. The HHC clinical care coordinator maintains an up-to-date PC register to facilitate a world-class service. Additionally, the HHC team provides subcutaneous infusions at home for cancer patients with moderate to severe pain in collaboration with a palliative consultant.

Telecare services in HHC were officially introduced in 2020 as part of digital transformation efforts and in response to the COVID-19 pandemic. The aim is to enhance patient’s access to care while ensuring their safety by reducing unnecessary physical visits to acute hospital facilities. Since then, telecare has become a vital part of HHC’s services, supporting both routine follow-ups and urgent consultations that are convenient for patients. This approach is also rewarding for nurses who deliver PC as they have more autonomy in the community and spend time in teleconsultations at the hospital with follow-up care, which offers variety in their working weeks. Safety is improved by reducing patients’ exposure to infections, especially for older adult or immunocompromised patients. Inequalities are reduced due to less waiting time and faster access to cost-effective consultations with family involvement, continuity of care and patient education. Nurses update electronic patient records and can invite clinical colleagues virtually for consultations on telecare platforms. They also help patients with minor technical issues.

## Future prospects

In terms of the future, healthcare practitioners in PC and end-of-life care nursing will face increasing gender equality and ethical challenges (64) in an ageing society with more complex patient needs and ongoing SDOH considerations. PC nurses will need to specialize in geriatric nursing as people living longer will add to compassion fatigue (67). As burdens increase for family caregivers (56), hospice care nursing skills will become more important within competency frameworks.

There will also be a greater need for PC physicians (20) and for non-medical prescribing, which is not allowed in KSA. Nurse-led pediatric PC (76) will also be an interesting area to reduce the gaps in knowledge and how nurses develop greater autonomy in the Kingdom. There will be an increasing need for healthcare leadership occupied by women in the Kingdom (40) and support for senior women’s careers (43), including working mothers (77) and nurses’ quality of working life in different settings like primary care (60) to improve employee satisfaction (63).

To bridge the gaps between current competencies and Vision 2030 aligned to global benchmarks, there needs to be a focus on improving core competencies amongst PC nurses (78) at different levels (79), especially in hospice care (80) and primary care (81). Attention to competencies in using artificial intelligence (82) will also be significant.

## Policy recommendations

Successful implementation of Saudi Vision 2030 healthcare reforms in the Ministry of Health’s last phase of life model of care relies on improving PC. This requires workforce competency and retention to reduce shortages and effective line management to reduce attrition and systems such as workforce engagement, planning, communications and support to deliver healthcare quality. We propose three specific recommendations related to equity, compassionate human resources and communications to bridge the gaps between Vision 2030 and practices on the ground related to pay equity, improving nursing competencies, enhancing compassionate organizational support, and creating a better sense of meaning in the workplace.

i. Gender and race pay equity

First, discrimination against nurses from developing countries (26) who form the backbone of the workforce is not conducive to promoting inclusive workplace cultures of caring for staff who care for members of the Saudi population with life-limiting diseases. These nurses are forced to take on overtime, which adds to the stress of working in emotionally sensitive conditions that stretch them physically, socially, spiritually and psychologically and shows a lack of compassion.

ii. Human resource for workforce health

Second, to ensure a healthy workforce of PC nurses, compassionate human resource policies and practices must reflect the values of a caring profession. This includes evidence-based induction programs, continuous professional development with support for personal growth in terms of knowledge and career progression, and the implementation of telehealth to enrich jobs. Skills are needed using telehealth to provide access to remote and marginalized communities and to compensate for nursing staffing shortages.

iii. Professional and public communication campaigns

Finally, the image and understanding of nursing and PC need to be improved as the demand for services grows. We recommend that the Ministry of Health, Saudi Nurses Association, and Saudi Commission for Health Specialties adopt corporate communication plans to educate healthcare workers and public members about inequality challenges related to accessing high-quality PC services. They need to train nurses, other healthcare practitioners, and service users to understand its value. It is imperative to increase respect for nurses and to understand the benefits of PC as a career and service.

## Conclusion

Self-evidently, as Campbell et al. (59) state, there is no health without a workforce. Furthermore, high-quality care requires a healthy nursing workforce (83) with fair and supportive working conditions to reduce stress levels. As healthcare reforms in KSA seek to raise the social determinants of health for local populations, they need to be accompanied by interventions to raise the social determinants of health for healthcare professionals.

The need for this study is underpinned by the substantial demand predicted in KSA for PC services for both cancer and non-cancer patients (9). It is also necessary to provide community-based palliative telehealth, which can enhance opportunities for improving the quality of nurses’ working lives and trusting relationships (84). Many terminally ill patients prefer to die at home (4) or in a hospice, and not in an acute hospital.

We argue that improving human resources for health is an important determinant for the quality of PC healthcare services delivered to populations in KSA in the context of nursing shortages and growing demand for PC services. Despite a focus on Saudisation, KSA continues to rely on expatriate nurses, especially in less attractive and stressful specialisms like PC, which require high levels of compassion. Further research might explore the challenges in pediatric PC. The maldistribution of health workers in rural and remote regions highlights socioeconomic disadvantages and digital divides, which are mirrored in the nursing healthcare workforce that serves these populations. Inequitable working conditions can affect healthcare workers’ motivation and satisfaction with their psychological status and performance which in turns affects patient care and satisfaction.

The availability of PC in KSA remains inequitable for many patients with serious chronic illnesses as services are patchy throughout the Kingdom, and there are insufficient qualified and experienced PC nurses. This review is important, therefore, to highlight key challenges and opportunities for change. Uniquely, we argue for improvements to workforce inequalities and attending to social determinants of health for PC nurses by directly linking to the potential impact on improving population health SDOH. If PC nurses feel that they are supported in compassionate cultures to sustain their own wellness and decent living and working conditions, they are likely to turn over their jobs less frequently and to be able to attend to the wellbeing of patients and their families. Hence, this study is a compelling, socially relevant issue in ageing societies with increasing PC demands.

Women in KSA continue to face complex, intersectional challenges and discrimination in a context where PC remains problematic and, at times, controversial. It is clear from the extant literature that the tensions between female migrants, lived experiences of care work in KSA are further marked by the inherent difficulties of PC, which requires high levels of emotional labor in a precarious, gendered labor market (32) marked by Saudisation (*nitaqat*), the replacing expatriates with Saudi workers (61). Furthermore, we argue that the strategy implementation gap in public sector healthcare in terms of equitable income and social protection of female PC nurses in KSA contributes to inequitable end-of-life care for patients and SDOH inequalities (7) for nurses themselves and society more broadly. A paradigm shift is required surrounding managing, rewarding and treating female expatriate PC nurses, a key factor outlined in the WHO (58) global strategy on human resources for health 2030.

Whilst large-scale efforts are being made to expand and develop nursing numbers and provision in the Kingdom, there is still a long way to go in providing equitable access to healthcare and PC. Additional policy efforts to reduce urban and rural imbalances in care remain imperative despite the official launch of Seha Virtual Hospital in 2022. If KSA is to succeed in addressing national policy-implementation gaps in healthcare transformation alongside Saudisation, inequalities need to be reduced. Policies in practice must include consideration of minimizing disparities in care provision between rural and urban areas. Organizations must provide training and remuneration packages to expatriate nurses which are equitable with that of their Saudi counterparts. Terms and conditions should be based on job descriptions and competency frameworks and not unfairly based on nationality. Nurses from developing countries are paid the least, although the Saudi healthcare system would collapse without them. Indeed, if the Saudi Society for Palliative Care is to be successful in its mission and aim of introducing “palliative medicine in our society in the right context and to unify the efforts of palliative medicine specialists to improve the services available to patients” (85), equity between migrant and home national nurses must be prioritized.

## Data Availability

The original contributions presented in the study are included in the article/supplementary material, further inquiries can be directed to the corresponding author/s.

## Footnotes

^1^The PRISMA 2020 flow diagram template is distributed in accordance with the terms of the Creative Commons Attribution (CC BY 4.0) license, which permits others to distribute, remix, adapt and build upon this work, for commercial use. See: https://creativecommons.org/licenses/by/4.0/; Source: https://www.prisma-statement.org/prisma-2020-flow-diagram (Retrieved 24/01/2025).
