# Correction: A narrative review of nursing in Saudi Arabia: prospects for improving social determinants of health for the female workforce

**DOI:** 10.3389/fpubh.2025.1694579

**Published:** 2025-10-06

**Authors:** Julie Davies, Emily Yarrow

**Affiliations:** ^1^Brunel Business School, Brunel University of London, Uxbridge, United Kingdom; ^2^Business School, Faculty of Humanities and Social Sciences, Newcastle University, Newcastle upon Tyne, United Kingdom

**Keywords:** gender equality, gender equity, healthcare, nursing, palliative care, Saudi Arabia

Author Steven Callaghan was erroneously included as an author in the published article. The correct author list reads:

Julie Davies and Emily Yarrow

The Author Contributions Statement has been corrected to read:

JD: Writing – original draft, Writing – review & editing. EY: Writing – original draft, Writing – review & editing.

The original version of this article has been updated.

